# Supplementary figures and images for: DSCR1 deficiency ameliorates the Aβ pathology of Alzheimer’s disease by enhancing microglial activity
Source: Life Sci Alliance. 2022 Nov 30;6(2):e202201556. doi: 10.26508/lsa.202201556 (PMC9713304; doi:10.26508/lsa.202201556)

## Short exposure

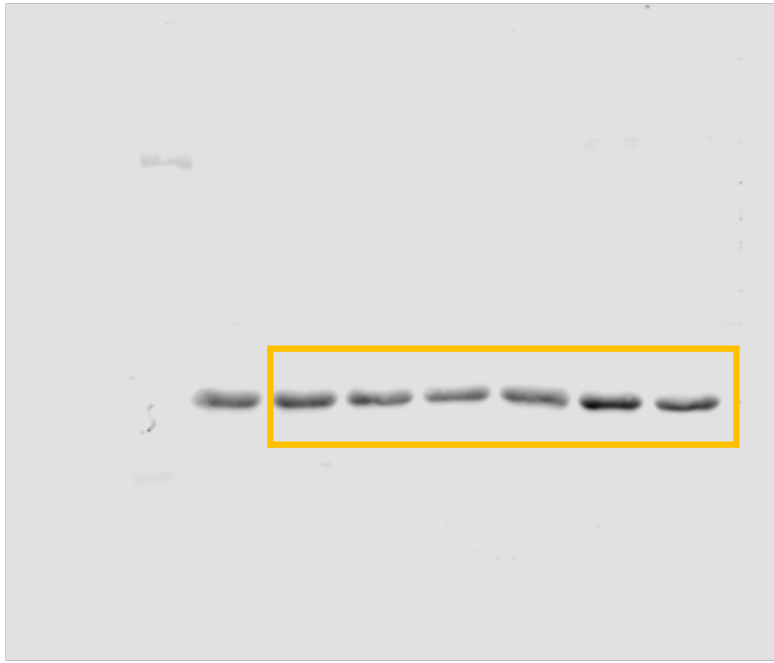

IB: Gapdh

(kDa)

## Long exposure

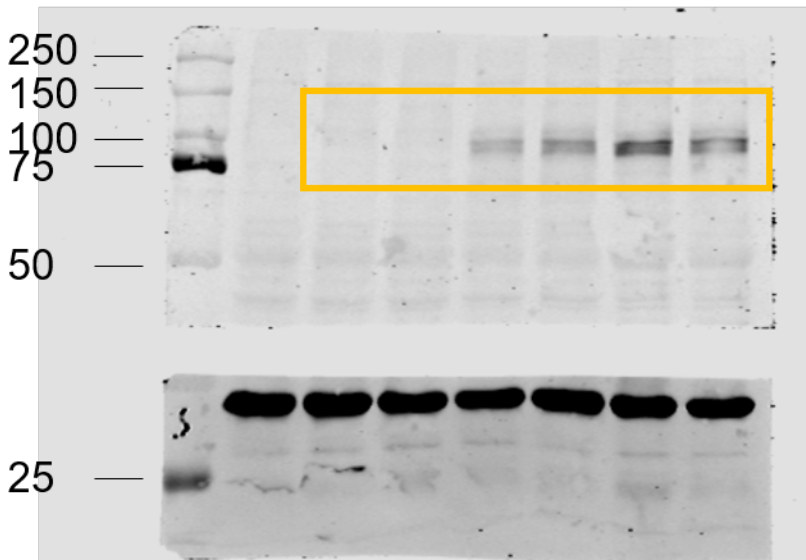

IB: hAPP

IB: Gapdh

Supplement: Supplementary file 1 [file LSA-2022-01556_SdataF2.1.pdf]

Short exposure

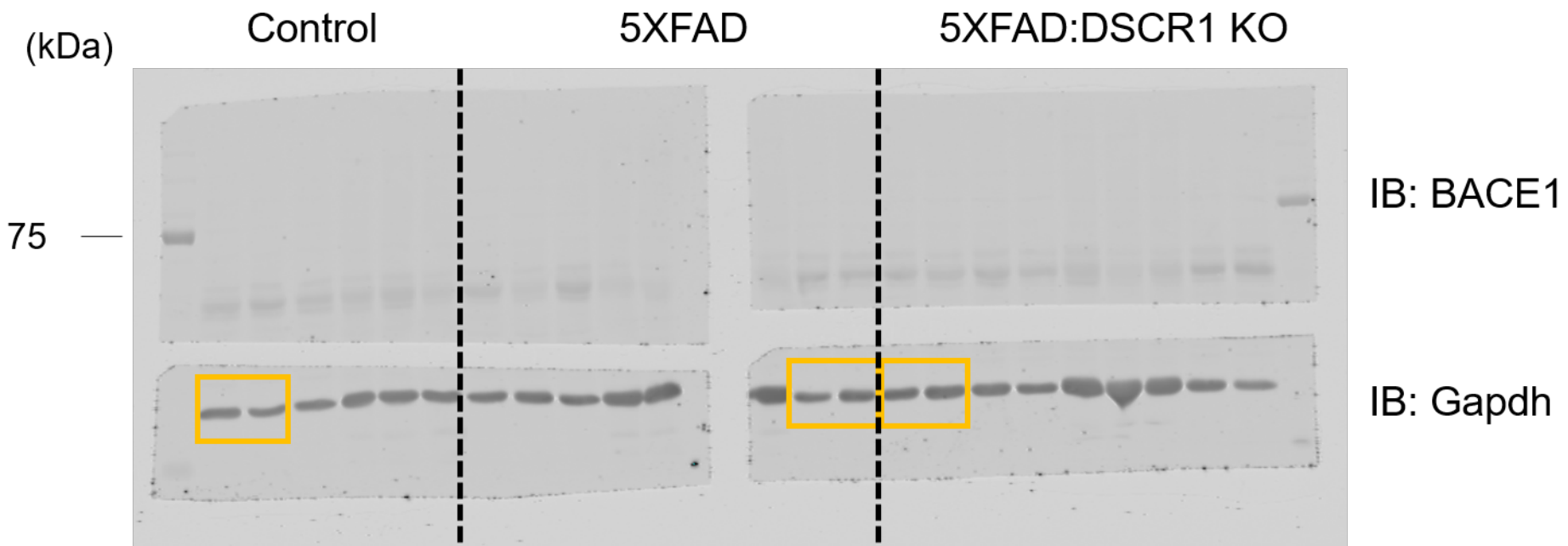

Long exposure

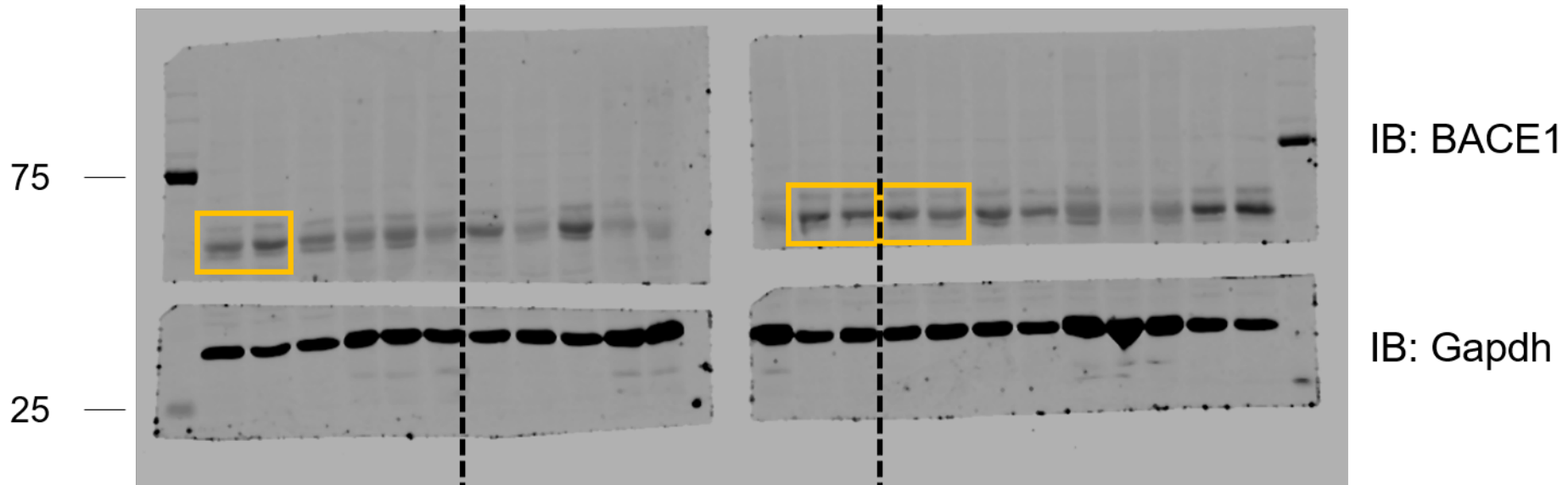

Supplement: Supplementary file 2 [file LSA-2022-01556_SdataF2.2.pdf]

5XFAD

5XFAD:DSCR1 KO

(kDa)

250 —

150 —

100 —

75 —

50 —

37 —

25 —

IB: hAPP

IB: Gapdh

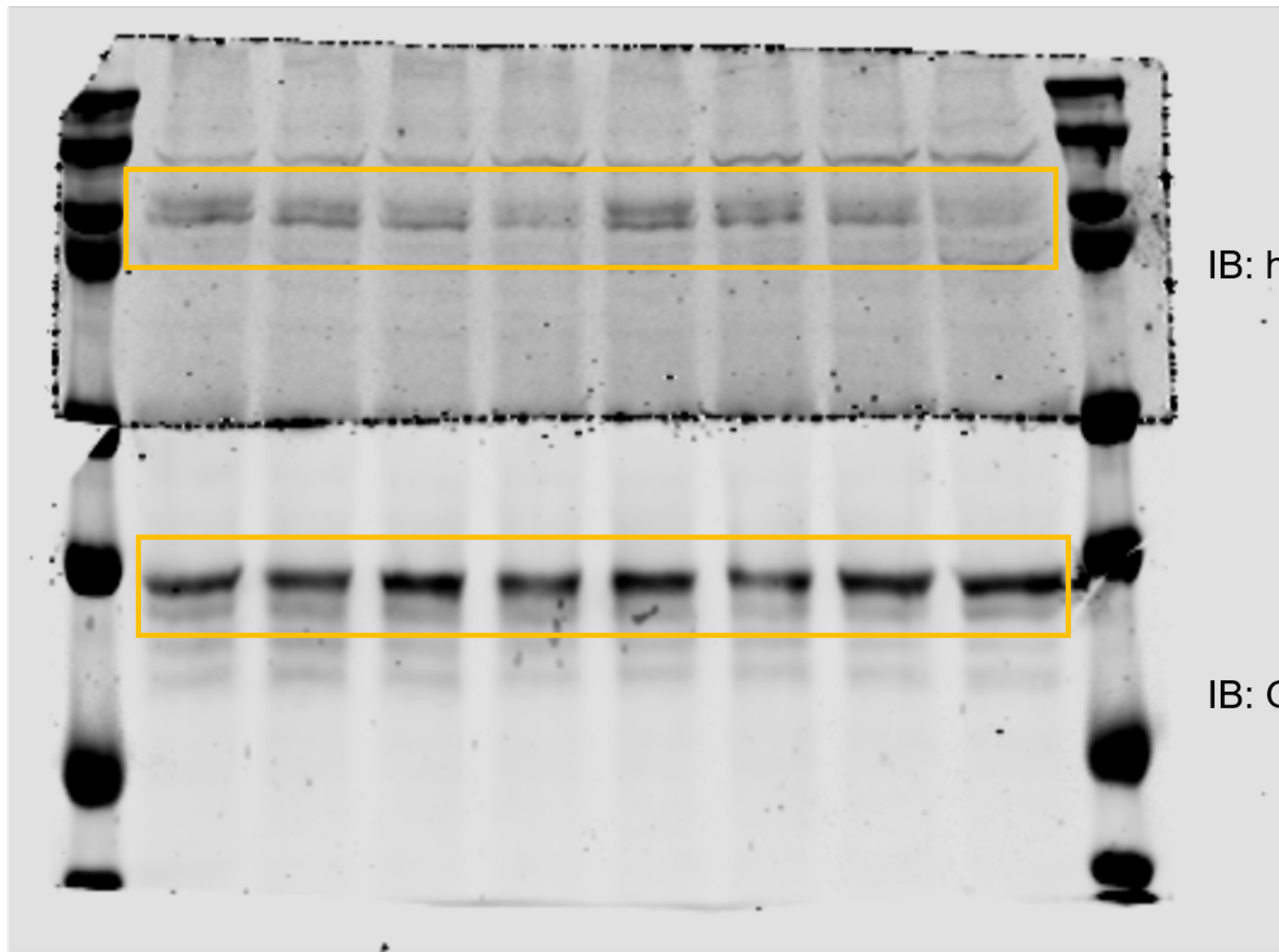

Supplement: Supplementary file 3 [file LSA-2022-01556_SdataFS1.pdf]

Short exposure

WT

DSCR1 KO

(kDa)

250  
150  
100  
75

IB: LAMP1

50  
37  
25

IB: Gapdh

Long exposure

(kDa)

250  
150  
100  
75

IB: LAMP1

50  
37  
25

IB: Gapdh

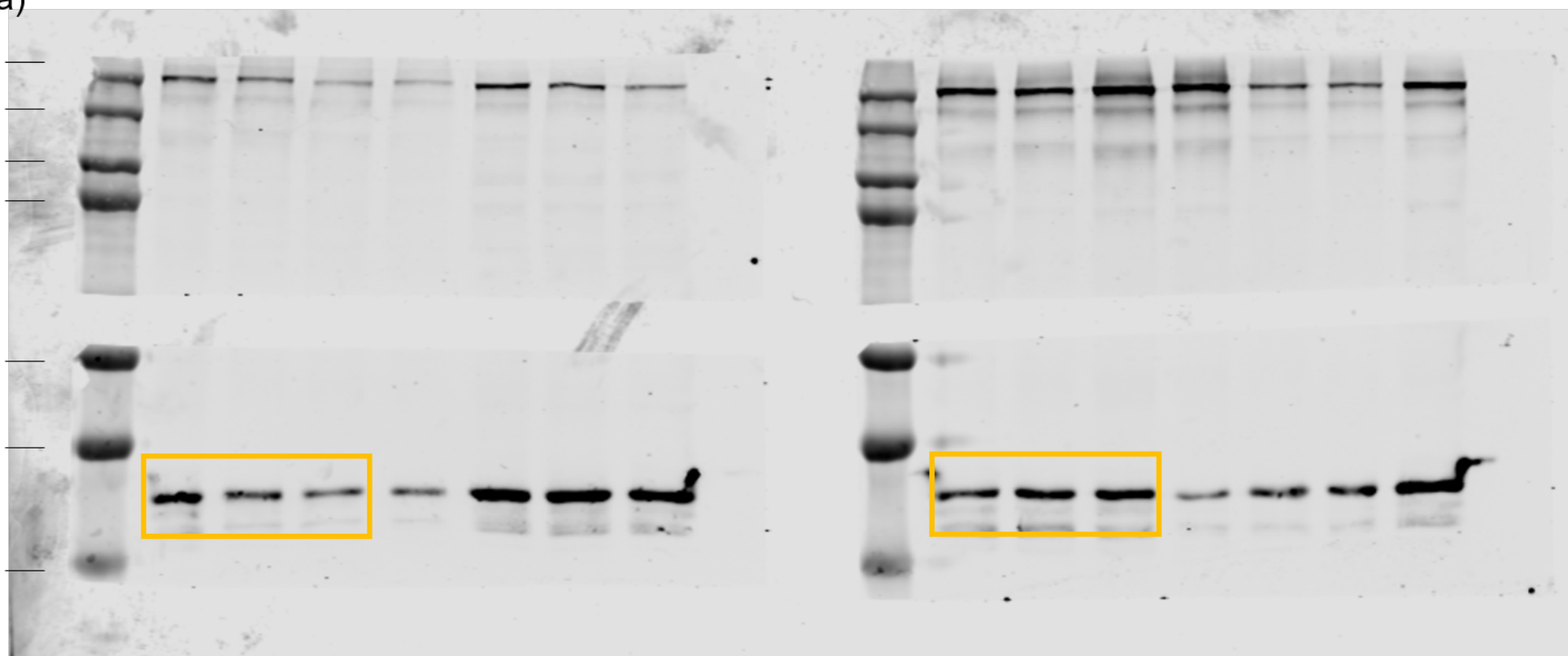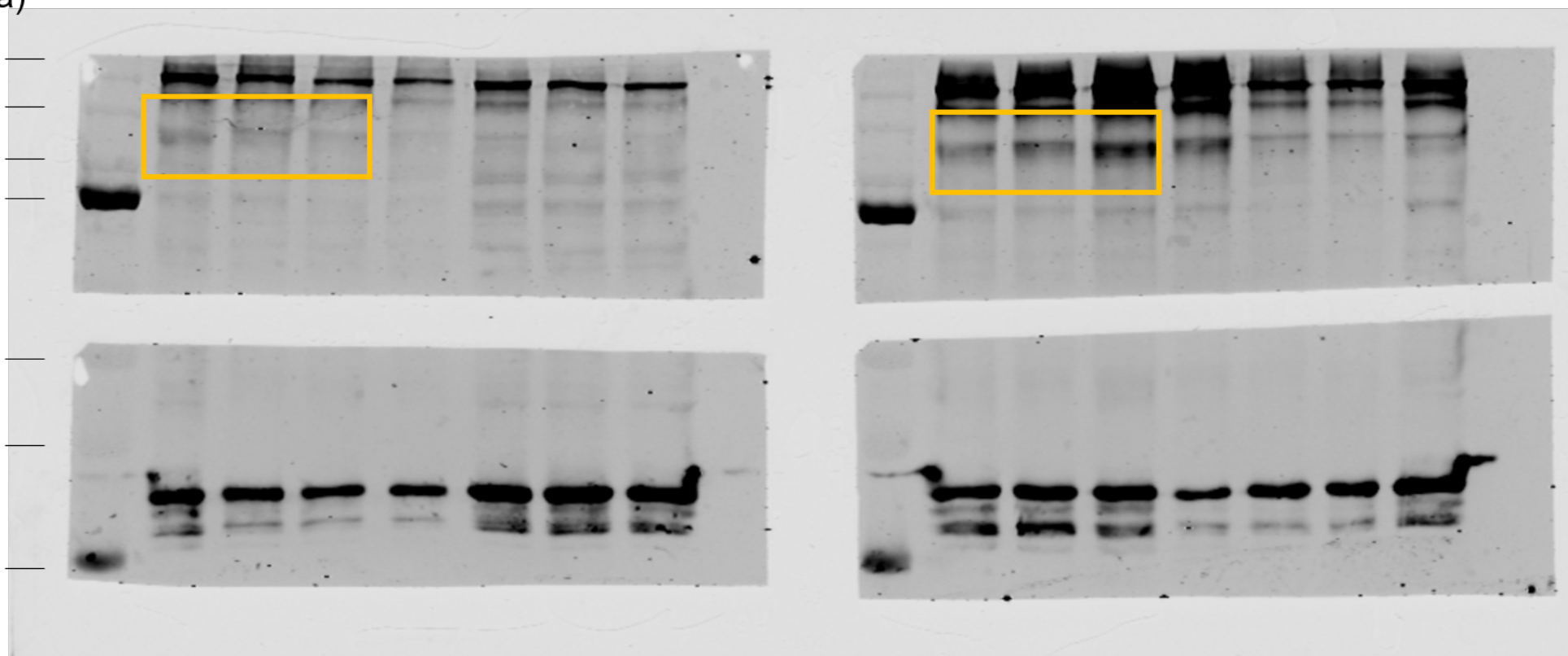

Supplement: Supplementary file 4 [file LSA-2022-01556_SdataF4.pdf]
